# The role of large language models in the peer-review process: opportunities and challenges for medical journal reviewers and editors

**DOI:** 10.3352/jeehp.2025.22.4

**Published:** 2025-01-16

**Authors:** Jisoo Lee, Jieun Lee, Jeong-Ju Yoo

**Affiliations:** 1 Department of Internal Medicine, Soonchunhyang University Bucheon Hospital, Bucheon, Korea; 2 Division of Gastroenterology and Hepatology, Department of Internal Medicine, Soonchunhyang University Bucheon Hospital, Bucheon, Korea; Hallym University, Korea

**Keywords:** Peer review, Large language models, Generative artificial intelligence, ChatGPT, Republic of Korea

## Abstract

The peer review process ensures the integrity of scientific research. This is particularly important in the medical field, where research findings directly impact patient care. However, the rapid growth of publications has strained reviewers, causing delays and potential declines in quality. Generative artificial intelligence, especially large language models (LLMs) such as ChatGPT, may assist researchers with efficient, high-quality reviews. This review explores the integration of LLMs into peer review, highlighting their strengths in linguistic tasks and challenges in assessing scientific validity, particularly in clinical medicine. Key points for integration include initial screening, reviewer matching, feedback support, and language review. However, implementing LLMs for these purposes will necessitate addressing biases, privacy concerns, and data confidentiality. We recommend using LLMs as complementary tools under clear guidelines to support, not replace, human expertise in maintaining rigorous peer review standards.

## Graphical abstract

[Fig f2-jeehp-22-04]

## Introduction

### Background/rationale

#### Evolution and current challenges of peer review

Peer review is the process through which independent experts evaluate scientific work for quality, novelty, and originality. Peer review began in 1665 at the Royal Society of London and has evolved over time to become the gold standard for scientific validity and integrity [1]. This process is particularly important in the medical field, where research findings can directly affect patient care and treatment outcomes [2]. Despite its fundamental role, the traditional peer review system has faced challenges due to its rather time-consuming nature, as reviewers have been swamped by the recent expansion of the research landscape in medicine. The exponential increase in publications—up by 47% from 2016 to 2022—has outpaced the expansion of practicing scientists [3]. This imbalance has created a substantial burden on the peer review system. According to a 2024 survey by Wiley, 70% of researchers take at least 4 days to complete their peer review of an article, and 62% of respondents cited time constraints as their biggest challenge [4].

#### The advent of generative AI in academia

Since the release of ChatGPT by OpenAI in November 2022, generative artificial intelligence (AI) has transformed numerous aspects of daily life. Generative AI is now widely used in education, business, healthcare, and other fields, and academic writing is no exception. Researchers have increasingly utilized AI tools for their scholarly work, such as literature reviews [5], data analysis [6], and manuscript writing [7].

This technological advancement may reduce the time and resources required for peer review and improve efficiency. However, the academic community has raised concerns about the impact of utilizing AI on scholarly integrity and review quality. Given these considerations, a thorough analysis of integrating generative AI into the peer review process is necessary.

### Objectives

The objective of this review is to analyze large language models (LLMs) in the peer review landscape, including their application and performance in scientific publishing. We analyzed both the benefits and challenges of integrating generative AI into the peer review process, and suggested future directions.

## Current status of generative AI in peer review

### Prevalence of applying generative AI in peer review

Various sources have demonstrated widespread adoption of generative AI in academic workflows. A survey of 3,838 postdoctoral researchers revealed that 31% of responders used generative AI primarily for text refinement (63%) [8]. According to a *Nature* survey of 1,659 researchers [9], 11% of responders considered expediting peer review as the primary benefit of generative AI, with 24% expecting this advantage to become the main function of generative AI in the future. However, some research claims otherwise; a study by Ng et al. [10] in 2024 reported that 44% of researchers believed that AI chatbots are not helpful for assisting with peer review, and 67% had never used AI chatbots to critique the works of other researchers.

These mixed survey results raise questions about the actual application of generative AI in peer review. Despite various studies exploring the actual usage of generative AI in academic settings, the results vary widely due to hindrances in differentiating LLM-generated reviews. Detecting LLM-generated reviews is difficult due to 2 main obstacles: (1) authors often do not disclose their use of AI tools, and (2) current detection methods, even by human reviewers, are still unreliable due to high false positive or negative rates [11]. Nevertheless, recent studies have provided empirical evidence on the extent of AI use in peer review. A commercial LLM detection service estimated that at least 15.8% of reviews for an AI conference were written with AI assistance [12]. Liang et al. [13] developed a corpus-level detection model based on word distribution patterns and demonstrated LLM modification rates of 6.5% to 16.9% in AI conference peer reviews. In contrast, a similar analysis of journals in the *Nature* journals showed no significant evidence of LLM-based modifications, suggesting that the use of AI tools varies widely across academic disciplines.

### Current performance of LLMs in the peer review process

Recent studies evaluating LLM capabilities in peer review tasks have shown mixed results across different assessment methodologies. These assessment approaches can be broadly categorized into 2 main types. The first approach focuses on review generation tasks, and the quality of these LLM-generated reviews is evaluated through various metrics including similarity to human reviews, specificity of feedback, and overall helpfulness. The second approach evaluates the error detection capabilities of LLM by artificially introducing errors into manuscripts and assessing its ability to identify and critique these flaws. This method provides a more controlled environment for evaluating its critical analysis capabilities in the context of peer review.

#### Performance in review generation tasks

In the medical field, Saad et al. [14] conducted a comparative analysis of reviews generated by GPT-3.5, GPT-4, and human reviewers for 21 medical manuscripts. The study revealed a limited correlation between AI-generated reviews and human reviews, and AI reviews showed a minimal association with the final acceptance rate. These findings suggest that LLMs face significant limitations in effectively evaluating medical research papers.

In contrast, Liang et al. [15] reported more encouraging results from a large-scale study involving *Nature* portfolio journals and AI conference papers. They found that the overlap between GPT-4-generated reviews and human reviews was comparable to the overlap between 2 human reviewers. Amongst the 308 researchers in the study, 57.4% found feedback by GPT-4 helpful, and 82.4% rated its performance superior to at least some human reviewers. D’Arcy et al. [16] proposed a multi-agent system approach, in which multiple AI agents with specialized roles collaborate to generate reviews, similar to the panel of human reviewers in traditional peer review systems.

Compared to the single-agent approach, the multi-agent method received high ratings for specificity (70.8% versus 40.0%) and overall (21.4% versus 8.6%) by human reviewers. Nonetheless, 38% to 48% of all comments generated by AI agents were rated as highly inaccurate, demonstrating the limitation of LLMs in peer review tasks.

#### Error detection capabilities

Studies evaluating the ability of LLMs to detect intentionally embedded errors have exhibited varying results. Liu and Shah [17] found that GPT-4 could identify 7 out of 13 intentionally inserted errors in research papers, performing comparably to human reviewers. However, Kadi and Aslaner [18] reported that GPT-4 struggled to detect major issues, such as mismatches between titles and contents, and disproportionately focused on minor typographical errors in 15 short medical articles.

LLMs demonstrated stronger capabilities in detecting language-related errors. Lechien et al. [19] focused specifically on linguistic accuracy, testing the ability of GPT-4 to review papers written by non-native English-speaking otolaryngologists. The results were favorable, with GPT-4 successfully identifying 83.7% of grammatical errors. These results suggest that LLMs could be particularly useful for improving the linguistic quality of academic manuscripts, especially for non-native English speakers.

### Factors affecting LLM performance

Several factors including drastic improvements in LLM technology, variations in prompting strategies, and discipline-specific limitations affect LLM performance and evaluation. Thanks to the rapid evolution and continual changes in LLM technology, the timing of studies vastly impacts study outcomes. For instance, there is a significant difference in performance between GPT-3.5 and GPT-4, which were released within a period of 1 or 2 years. These rapid and unpredictable shifts in LLM capabilities prevent researchers from drawing definitive conclusions on the effectiveness of LLMs in peer review.

Variation in prompting strategies across studies emerges as another crucial factor affecting LLM performance. For instance, while Saad et al. [14] used simple prompts requesting 3 advantages and disadvantages for each manuscript, other studies adopted more sophisticated methods, such as adopting specific reviewer personas or structured evaluation frameworks. Santu et al. [20] systematically investigated this effect in their meta-review generation experiment, comparing 4 levels of prompt complexity. Performance improved significantly from basic (level 1) to moderately-structured prompts (level 2), but high complexity prompts (levels 3 and 4) showed diminishing returns. This suggests the need for an optimal balance in prompt design for peer review.

Interestingly, LLMs consistently underperform in evaluating medical research papers compared to other fields. This trend is supported by the analysis of Thelwall [21] of 34 academic disciplines, which revealed that clinical medicine was the only field where the quality scores of GPT-4o mini displayed a negative correlation with the actual paper quality. Among the three medical studies mentioned earlier [14,18,19], all except one study[19]—focusing on language correction—reported that LLM performance was generally unsatisfactory. focusing on language correction. This distinctive pattern in clinical medicine may arise from several factors. First, medical papers often use a characteristically cautious tone due to potential health impacts. Also, most clinical medicine studies rely heavily on statistical results and precise numerical data, which LLMs struggle to interpret effectively. These domain-specific challenges suggest that applying LLMs in medical peer review may require specialized approaches or additional safeguards, unlike in other academic disciplines.

### Implications

Thorough examinations of LLM capabilities have allowed researchers to understand the supplementary role of LLM in scientific writing. LLMs demonstrate considerable proficiency in language-related tasks, such as identifying grammatical errors, generating structured feedback, and detecting linguistic inconsistencies.

However, their strong focus on linguistic elements presents an unexpected limitation: their increased sensitivity to language patterns may hinder their evaluation of fundamental scientific analyses in research publications. This bias toward linguistic presentation can result in the misinterpretation of cautiously worded statements, especially in fields like clinical medicine. LLMs may struggle to differentiate between appropriately cautious writing and a lack of research confidence, which may compromise their efficacy in assessing such articles.

These findings suggest that LLMs are best positioned as complementary tools rather than automatic, standalone reviewers in the peer review process. Their strengths in language-related tasks are valuable for initial screening and basic feedback, but their limitations in assessing scientific validity beyond linguistic presentation demand ongoing human supervision. This is especially crucial in clinical medicine, in which interpreting statistical data and evaluating clinical significance require deep domain expertise that current LLMs have not yet mastered.

### Integrating LLMs into the peer review process

**Fig. 1.**
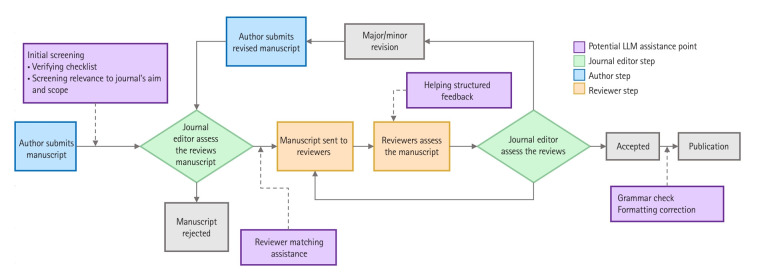
Peer review process with large language model (LLM) integration. Conventional peer review process and the potential LLM integration points (highlighted in purple).

The traditional peer review process is resource-intensive and time-consuming, presenting opportunities to improve efficiency and accuracy. Fig. 1 illustrates potential points where LLMs can significantly improve efficiency and accuracy.

### Initial screening

Initial manuscript screening, a critical but time-consuming step in the editorial process, involves evaluating submissions for scope alignment, quality standards, and technical requirements. Applying LLMs in screening manuscripts by titles and abstracts has been discussed mainly in the context of systematic reviewing and guideline writing. A recent study using GPT-4 Turbo demonstrated high accuracy in the evaluation of titles and abstracts when employing specific prompt strategies [22]. Dennstädt et al. [23] evaluated various LLM models, reporting sensitivity rates from 81.93% to 97.58% and specificity of over 99.9%. Journal editors may utilize this technology to effectively screen manuscripts that correspond with the aim of their journals, thereby potentially decreasing the administrative burden associated with initial manuscript screening. Efficient pre-screening with LLMs enables reviewers to focus only on relevant manuscripts during the matching stage, optimizing the use of editorial resources.

### Reviewer matching

Reviewer matching is another potential application of LLMs in the peer review process. Identifying appropriate reviewers is a complex task for journal editors, involving multiple considerations such as aligning manuscript topics with reviewer expertise, screening for conflicts of interest, confirming availability, and ensuring diverse academic perspectives. By employing machine learning algorithms, LLMs can analyze patterns of reviewer preferences and past performances, optimizing the selection process and broadening the pool of available reviewers [24]. Farber [25] reported a 42% overlap between GPT-4-suggested reviewers and those manually selected by editors and 37% additional qualified reviewers identified by GPT-4, who were not initially considered. Notably, GPT-4 reduced reviewer selection time by 73%, from 45 to 12 minutes, demonstrating its potential to improve efficiency and identify experts across diverse fields.

### Helping with structured feedback

The current peer review system heavily relies on the subjective judgment of reviewers, hindering consistency and objectivity in review outcomes. With the rapid increase in academic submissions, reviewers often struggle to dedicate sufficient time to each review; sometimes, time pressure and anonymity have even led to unconstructive or aggressive comments. Once integrated into the review workflow, LLMs can efficiently assist in conducting structured, refined reviews. LLMs can help human reviewers by analyzing manuscripts against predefined evaluation criteria, ensuring that critical aspects are not overlooked. Additionally, LLMs can refine the tone of review reports, facilitating more constructive and less aggressive comments [26].

### Grammar and format checking

LLMs are highly effective tools for formatting and grammar correction. Even skeptics acknowledge the linguistic strengths of LLMs [18,27]. This is especially beneficial for non-native English-speaking researchers, enabling proficient writing and reducing linguistic bias in academic publishing [28,29]. By improving the linguistic quality of academic papers, LLMs may serve as valuable supplementary tools that improve the overall clarity and accessibility of scholarly communication.

### Key challenges

While LLMs can improve efficiency in tasks such as initial screening and structured feedback, it is essential to address a broad range of ethical and practical considerations—including potential language biases, as well as privacy and confidentiality concerns—in order to ensure the fairness and integrity of scientific publishing.

### Bias

Bias in LLM-assisted peer review represents a complex challenge that requires careful consideration and systematic management. Although traditional peer review already exhibits biases, such as geographical disparities in reviewer selection and language-based discrimination [30], the integration of LLMs may add further complexities to these challenges.

The primary challenge comes from the biases in data used in LLM training. Since these models are mainly trained on English-language academic texts from leading institutions, LLMs risk prioritizing dominant academic perspectives while underrepresenting research from non-English-speaking regions. However, after careful implementation, LLMs have the potential to mitigate some human biases, particularly those related to language. A European Research Council survey found that 75% of surveyees anticipated that generative AI could reduce language barriers in research by 2030. Beyond language, standardized LLM-assisted review protocols may also minimize other biases, such as preferences for institutional prestige, nationalities of authors or specific methodologies, promoting fairness and inclusivity in the peer-review process.

### Privacy and confidentiality

Privacy and confidentiality concerns are critical challenges in integrating LLMs into peer review processes, particularly in medical publishing. There are 3 key concerns: pre-publication data protection, model data retention, and healthcare-specific compliance requirements.

The primary risk involves the exposure of unpublished research data during the review process. Manuscripts processed through LLM systems are vulnerable to premature data disclosure, which could compromise the integrity of the blind review process and violate publication embargoes. Beyond immediate risks, LLMs also retain processed information in their training datasets, creating long-term vulnerabilities related to data security and the protection of intellectual property. Such risks for privacy breaches are particularly severe in medical manuscript reviews, in which manuscripts frequently contain sensitive patient information and clinical trial data. Many third-party LLM services rely on cloud-based processing systems with varying security protocols and data handling policies [31]. These vulnerabilities lead to additional complexity in maintaining compliance with healthcare data protection requirements [32].

## Recommendations

### For the academic community

Peer reviewers and editors need to have a clear understanding of LLM usage. Fears of LLMs, sometimes reflecting overestimations or underestimations, often stem from a lack of knowledge about their capabilities and limitations. Stakeholders must cultivate an accurate understanding of the capabilities and limitations of LLMs through regular workshops and accessible educational resources. Although a comprehensive understanding may be challenging due to the rapid development of LLMs, ongoing efforts must be made to keep current with their latest updates. As previously discussed, particular caution must be taken to maintain data security and confidentiality while managing sensitive research data or clinical information in LLMs.

### For reviewers

Reviewers can utilize LLMs to optimize basic review tasks, such as grammar and format checking, allowing reviewers to focus on advanced aspects of peer review. These include critically analyzing the overall significance, novelty and impact of the research, distinguishing subtle differences in methodologies or findings, and providing field-specific insights.

Reviewers must use LLMs responsibly and avoid over-reliance. Analyses or suggestions provided by LLMs should be critically assessed, especially those pertaining to statistical analyses or complex methodologies. Human reviewers should treat LLMs solely as tools, remaining accountable for their proper use by carefully verifying the accuracy and reliability of LLM outputs and reporting any potential issues to the editor.

### For editors

Editors should establish clear guidelines for LLM usage to ensure quality and integrity enhancement—not impairment—of academic communication. While many journal-specific guidelines had rarely addressed generative AI by early 2023—or mainly focused on writers, had they addressed generative AI—there has lately been a notable progress. A study by Ganjavi et al. [33] showed a 25% increase in generative AI-related guidelines among the top 100 medical journals between March and October 2023, with growing attention to reviewer usage. However, many non-top-tier journals still lack relevant guidelines [27]. Additionally, there is a considerable variation in guidelines across journals and publishers and in terms such as generative AI, LLMs, and AI, which are often used inconsistently. Editors must address these issues by providing reviewers with clear guidance.

It is also essential to continuously evaluate and adjust the impact of LLM usage in the peer review process. Over-reliance on LLMs in reviews may urge researchers to shape their work based on LLM-based evaluation criteria, introducing potential biases that need to be guarded against. Researchers must be encouraged to transparently disclose, rather than conceal, their use of LLMs.

## Conclusion

We identified 4 potential integration points for LLMs in the peer review process: initial screening, reviewer matching, structured feedback assistance, and grammar and format checking. While challenges such as bias, privacy, and security in regard to unpublished data must be addressed, LLMs hold great potential to complement human expertise and enhance the efficiency, equity, and inclusivity of peer review. The effective and ethical application of LLMs relies on not only the technology itself, but also the expertise, critical judgment, and caution of researchers. Therefore, LLMs should be viewed as complementary tools, not as replacements for human expertise. To manage LLMs responsibly and transparently, it is essential for all members of the academic community to engage openly in discussions, share experiences, and collectively develop best practices for implementing LLMs in scientific writing. Editors and reviewers should establish clear guidelines for AI use, ensure transparency and focus human resources on complex tasks that require critical analysis and specialized knowledge.

## Figures and Tables

**Figure f2-jeehp-22-04:**
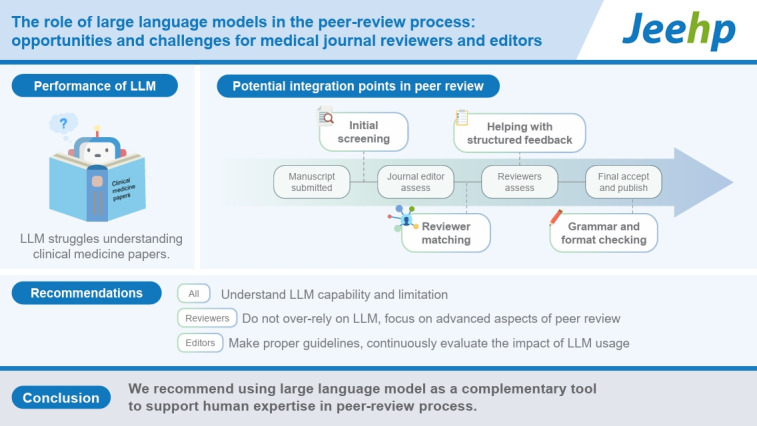


## Supplementary materials

Supplement 1. Audio recording of the abstract.
